# Rethinking Peer Review – Engaging Early Career Researchers and Promoting Disciplinary Diversity

**DOI:** 10.1007/s00192-026-06673-w

**Published:** 2026-04-29

**Authors:** Baerbel Junginger

**Affiliations:** 1 Charité Universitaetsmedizin Berlin, Berlin, Germany; 2 Evidenced Based Pelvic Floor Education Junginger, Gerstetten, Germany


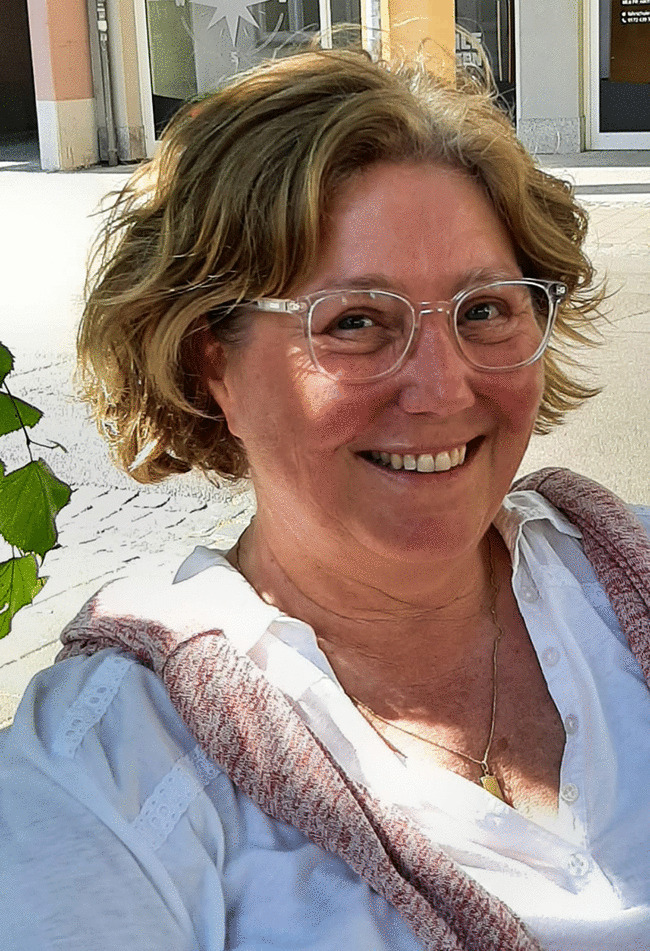


The scientific publication system is evolving rapidly. The number of manuscript submissions continues to grow, as does the diversity of journals and publication formats. Open Access has significantly increased the visibility of research and is now an indispensable part of modern science. At the same time, I, like many colleagues (Editors), have experienced an increasing challenge: it is becoming progressively harder to recruit enough qualified reviewers. Peer review remains the cornerstone of scientific quality assurance, yet this foundation is under mounting pressure. Review timelines are lengthening, requests go unanswered, and the responsibility falls on comparatively few shoulders. For me, the question is no longer *whether* we should act, but *how*.

My perspective on this issue is closely linked to my own professional journey. IUGA has enabled me to experience the publication system in various roles: initially as an author, later as a member of scientific committees (three years on the Educational Committee, six years on the Scientific Committee), as a reviewer, Editorial Board Member, and ultimately as an editor. Most of the time, I was the only physiotherapist. Now the editor, I am the first in my profession to hold this position. All my previous roles have made me realize that peer reviewing means learning continuously. Reviewing manuscripts has positively shaped my own writing, sharpened my eye for methodological quality, and taught me to present arguments more precisely and critically. For me, preparing peer reviews has never just been a task; it has also always been a learning space. At the same time, my engagement has always been motivated by a clear purpose: to bring the perspective and topics of physiotherapy more prominently into scientific discourse—at regional symposia and annual meetings hold by IUGA, and in the IUJ.

Early on in my career as an author, I noticed that physiotherapy journals are not too much interested in pelvic health. I also realized that many reviews in specified journals—frequently provided by colleagues from medicine (the majority in many professional societies)—did not fully capture the specific concerns of physiotherapy. While the feedback was often methodologically sound, it frequently missed thematical alignment with my articles’ content. More content-focused questions could have significantly enhanced the quality of the manuscripts. This experience did, however, not discourage me; it motivated me! It highlighted how essential it is to include diverse disciplinary perspectives in the peer review process. Scientific quality is built not only on methodological rigor but also on content expertise. I therefore believe that a vital next step is to involve more physiotherapists actively in reviewer pools, editorial boards, and scientific committees—not as a counterbalance to other disciplines, but as a necessary complement. This also applies to nurses, midwives and other professionals working in the field of pelvic health.

At the same time, it is becoming increasingly clear that the reviewer pool must be expanded: doctoral candidates and advanced master’s students already possess substantial methodological knowledge and subject-specific expertise. Yet they are rarely integrated into the peer review process. Programs such as “Peerspectives” at Charité – Universitätsmedizin Berlin demonstrate a different approach: early-career researchers receive targeted training and produce supervised reviews. Advantages have been demonstrated, even if currently only as preprints [1]. Similar models exist within structured doctoral programs at other universities. These initiatives confirm my own experience: peer review is teachable, and early involvement strengthens both individual competence and the system as a whole. It also helps potential authors to write in a way they know would be more likely to be accepted and reviewed positively / with little changes…

Engaging early-career researchers is far more than a pragmatic response to increasing workloads. It is an investment in the quality of scientific training. Those who review read differently—more critically, more attentively, and more systematically. They learn to question arguments, evaluate evidence, and assess manuscripts holistically. These skills are essential, especially at a time when generative AI raises new questions about independent scholarly work. At the same time, integrating young scientists offers the opportunity to embed disciplinary diversity early in the system, particularly for fields such as physiotherapy.

Of course, opening peer review to early-career researchers must not compromise quality. Based on my experience, supervision is key. Mentored review processes, clear guidelines, and structured training provide the necessary framework. They allow junior researchers to contribute without reducing the quality of reviews—on the contrary, the interaction between experienced and junior reviewers often produces more nuanced assessments (https://peerspectives.premier.charite.de/index.php/Peerspectives).

The expanding publication landscape reflects an active, dynamic scientific community. Yet it also demands new structures. For me, it is clear: systematically engaging early-career researchers and promoting broader disciplinary diversity are not optional measures. They are necessary steps to ensure the quality and sustainability of peer review. I therefore encourage the following concrete actions: journals should establish structured programs for early-career reviewers (for example, within IUGA, the Journal Club and the Fellows, Trainees, and Early Career Professionals Committee could be extended to include physiotherapists or equivalent initiatives launched); submission platform should be adapted and both should be mentioned and listed: the fellow or young scientist and its experienced mentor/ supervisor as a team that performed the review together; universities should integrate peer review into master’s and doctoral curricula, including credit points for training participation and completed reviews; senior researchers should actively mentor the peer review process, as in-person discussions with international colleagues in medicine and physiotherapy show differing perspectives; and professional societies and journal editors should actively work toward greater disciplinary diversity in reviewer pools and committees, including stronger inclusion of physiotherapy, nursing, and midwifery.

Looking back on my own journey, I know that I have learned a lot about science in my role as a reviewer. Perhaps this is where the answer lies: peer review not only as a duty but as a learning and developmental space to simultaneously relieve the system but also strengthen it sustainably.

If peer review is the foundation of scientific quality, then we need to invest in how it is supported in the future. This includes creating clear opportunities for early-career researchers to take part in peer review, integrating training into academic programs, and recognizing this work as part of scientific development. Mentorship should also be an important part of the process. At the same time, it is important to broaden participation and include a wider range of professional perspectives, including nurses, physiotherapists, midwives, and others working in the field. These steps will help ensure that peer review remains strong and sustainable as scientific publishing continues to evolve.

## Data Availability

No data.

## References

[1] Rohmann JL, Wuelk N, Rubarth K, Grillmaier H, Abdikarim I, Simoes ML, Schroter S, Piccininni M, Kurth T, Glatz T. Engaging doctoral students in peer review: a pre-post study evaluating the effectiveness of the “Peerspectives” course on review quality, knowledge and skills. medRxiv. 2025;2025–02. 10.1101/2025.02.11.25322060.

